# Metabolomic Profiles Associated With Difference in Muscle Protein Degradation Between Fast‐ and Slow‐Growing Chicks During the Neonatal Period

**DOI:** 10.1111/asj.70169

**Published:** 2026-03-31

**Authors:** Saki Shimamoto, Hanae Katayama, Daichi Ijiri, Shinobu Fujimura, Kazuki Nakashima, Shinya Ishihara, Shozo Tomonaga, Akira Ohtsuka, Miyu Kamimura

**Affiliations:** ^1^ Department of Animal Science and Welfare, Joint Faculty of Veterinary Medicine Kagoshima University Kagoshima Japan; ^2^ Graduate School of Science and Technology Niigata University Niigata Japan; ^3^ Department of Life and Environmental Science Kagoshima Prefectural College Kagoshima Japan; ^4^ Graduate School of Applied Life Science Nippon Veterinary and Life Science University Tokyo Japan; ^5^ Graduate School of Agriculture Kyoto University Kyoto Japan; ^6^ South Kyushu Livestock Veterinary Center, Joint Faculty of Veterinary Medicine Kagoshima University Kagoshima Japan

**Keywords:** broiler, metabolomics, neonatal period, protein degradation

## Abstract

We aimed to identify metabolites and metabolic pathways involved in differences in muscle protein degradation in broiler chicks during the neonatal period. To this end, we performed gas chromatography–mass spectrometry‐based metabolomic analyses of plasma, pectoralis major muscle, and liver samples. Based on body weight gain from 1 to 5 days of age, 5‐day‐old broiler chicks (Ross308) were divided into three groups (slow‐, intermediate‐, and fast‐growing groups). First, we confirmed that muscle protein degradation levels were closely associated with body weight gain. Untargeted metabolomic analysis revealed that 4, 14, and 22 metabolites in plasma, muscle, and liver, respectively, were correlated with the body weight gain of chicks from 1 to 5 days of age (Pearson's correlation, |*r*| ≥ 0.4). Most metabolic pathways identified in plasma and muscle were involved in either protein or amino acid metabolism, while those in liver were related to tricarboxylic acid cycle and carbohydrate metabolism. Interestingly, the expression of genes encoding enzymes related to branched‐chain amino acid (BCAA) catabolism were higher in the slow‐growing chicks than in the fast‐growing ones. These results suggest that BCAA catabolism is important for muscle protein degradation levels and body growth of broiler chicks during the neonatal period.

## 1 Introduction

For decades, genetic selection in broiler chickens has increased their growth rate, improving feed efficiency, increasing breast muscle size and increasing weight (Havenstein et al. 2003a, 2003b; Siegel 2014). This selection had significant impacts on metabolic characteristics, mainly attributable to selective pressure on major metabolic organs including the liver and muscle (Scheuermann et al. 2003; Schmidt et al. 2009; Neeteson‐van Nieuwenhoven et al. 2013). Meanwhile, the development of integrated large‐scale rearing in the poultry industry has given rise to large individual differences in growth rates (Tickle et al. 2018).

Skeletal muscle mass is controlled through a delicate balance between protein synthesis and protein degradation (Russell 2010). Changes in the rate of protein degradation may contribute to either excessive muscle growth or muscle atrophy (Goldspink 1976; Goldspink and Goldspink 1977). In chickens, it has been considered that the muscle protein degradation levels dominantly affect muscle growth because fast‐growing strains and/or individuals show lower protein degradation levels than their slow‐growing counterparts (Hayashi et al. 1985; Maeda et al. 1987; Tomas et al. 1988). In addition, we previously reported that the plasma N^τ^‐methylhistidine (N^τ^‐MeHis) concentration, an index of muscle protein degradation, was significantly lower in fast‐growing than in slow‐growing chicks of the same breeding lines (Shimamoto et al. 2018). However, the reasons for individual differences in the levels of muscle protein degradation of chicks within a strain remain unclear.

Recently, metabolomics has been used to examine the relationship between growth stage and growth rate, the impacts of nutritional status, and the influence of pathological condition on metabolites in livestock (Tomonaga et al. 2022; Imaz et al. 2022; Xie et al. 2023; Indriani et al. 2024). In this study, to identify metabolites and metabolic pathways involved in differences in either muscle protein degradation or body growth of broiler chicks during the neonatal stage, gas chromatography–mass spectrometry (GC‐MS)–based metabolomic analyses were performed on plasma, pectoral muscle, and liver of chickens exhibiting different growth rates during this stage.

## 2 Materials and Methods

### 2.1 Animal Experiment 1

One hundred male chicks (Ross 308) at 1 day of age were supplied by a commercial hatchery (Kumiai Hina Center, Kagoshima, Japan). They were individually numbered and housed together in an electrically heated battery brooder, with ad libitum access to water and a natural‐ingredient diet free of animal protein. The feed composition was formulated according to the Japanese Feeding Standard for Poultry (2011), and the details are presented in Table 1. Body weight was measured at 1 and 5 days of age to calculate body weight gain during this period. After confirming a normal distribution of the growth data using the Shapiro–Wilk test (*p* = 0.23), chicks were ranked according to their body weight gain. At 5 days of age, 24 slow‐growing chicks (the bottom 25%) and an equal number of fast‐growing chicks (the top 25%) were selected based on their body weight gain: the slow‐growing group (25.43 ± 0.86 g) and the fast‐growing group (53.94 ± 0.67 g). From the remaining chicks that were not selected for either group, six were randomly chosen and euthanized by decapitation under carbon dioxide anesthesia, to examine the distribution of N^τ^‐methylhistidine (N^τ^‐MeHis) among the tissues and organs in 5‐day‐old chicks. All chicks were housed four per stainless steel cage until 7 days of age, with six cages per group. Measurements from chicks within a cage were averaged to obtain a single value per cage, which was used for statistical analysis (*n* = 6). Feed intake (FI) and body weight were recorded daily. All excreta samples were collected during the last 2 days of the experiment. At 7 days of age, all chicks were euthanized by decapitation under carbon dioxide anesthesia, and then blood samples and whole‐skeletal muscles were collected. The skeletal muscles throughout the body were excised as extensively as possible using ophthalmic scissors, following the method of Hayashi et al. (1985). The experimental protocols and procedures were reviewed and approved by the Animal Care and Use Committee of Kagoshima University (Approval Number 17A006).

**TABLE 1:** Composition and nutrient analysis of the basal diet in animal experiment 1.

Ingredients (g/100 g)		
Corn meal	57.90	
Soybean meal	34.00	
Corn oil	4.30	
CaHPO_4_	2.00	
CaCO_3_	0.66	
NaCl	0.50	
Mineral and vitamin premix^a^	0.50	
DL‐methionine	0.14	
Calculated analysis		
Crude protein (%)	20.0	
Metabolizable energy (Mcal/kg)	3.1	

^a^ Content per kg of the vitamin and mineral premix: vitamin A 90 mg, vitamin D3 1 mg, DL‐alpha‐tocopherol acetate 2000 mg, vitamin K3 229 mg, thiamin nitrate 444 mg, riboflavin 720 mg, calcium d‐pantothenate 2174 mg, nicotinamide 7000 mg, pyridoxine hydrochloride 700 mg, biotin 30 mg, folic acid 110 mg, cyanocobalamine 2 mg, calcium iodinate 108 mg, MgO 198,991 mg, MnSO_4_ 32,985 mg, ZnSO_4_ 19,753 mg, FeSO_4_ 43,523 mg, CuSO_4_ 4019 mg, and choline chloride 299,608 mg.

### 2.2 Animal Experiment 2

Eighty male chicks (Ross 308) at 1 day of age were purchased from a commercial hatchery (Onuma, Niigata, Japan). The chicks fed the experimental diet free of animal protein, which was formulated according to the Ross 308 Broiler Nutrition Specifications (Aviagen 2019), and the detailed composition is presented in Table 2. All other rearing conditions were identical to those described for the animal experiment 1 until 5 days of age. After confirming a normal distribution of the growth data using the Shapiro–Wilk test (*p* = 0.12), chicks were ranked according to their body weight gain. At 5 days of age, the chicks were divided into three groups based on their body weight gain from 1 to 5 days of age: a slow‐growing group (the bottom 15%; 19.5 ± 0.4 g), an intermediate‐growing group (the middle 15%; 34.0 ± 0.2 g) and a fast‐growing group (the top 15%; 48.5 ± 1.0 g). Eight chicks were randomly chosen from each of these groups. These chicks were euthanized by decapitation under carbon dioxide anesthesia, after which they were dissected to collect the pectoralis major muscle and liver. The pectoralis major muscle weight was measured, and then the pectoralis major muscle and liver were snap‐frozen in liquid nitrogen and stored at −80°C until use. Blood samples were collected in heparinized test tubes, which were promptly centrifuged at 5900 × g for 10 min at 4°C to separate plasma and then stored at −30°C until analysis. The experimental protocols and procedures were reviewed and approved by the Animal Care and Use Committee of Niigata University (Approval Number SA00940).

**TABLE 2:** Composition and nutrient analysis of the basal diet in animal experiment 2.

Ingredients (g/100 g)	
Corn meal	49.80
Soybean meal	39.90
Corn oil	4.30
CaHPO_4_	2.00
CaCO_3_	1.26
Corn gluten meal	1.20
NaCl	0.50
Mineral and vitamin premix^a^	0.50
DL‐methionine	0.34
Lys‐HCl	0.13
Threonine	0.08
Calculated analysis	
Crude protein (%)	23.0
Metabolizable energy (Mcal/kg)	3.0

^a^ Content per kg of the vitamin and mineral premix: vitamin A 90 mg, vitamin D3 1 mg, DL‐alpha‐tocopherol acetate 2000 mg, vitamin K3 229 mg, thiamin nitrate 444 mg, riboflavin 720 mg, calcium d‐pantothenate 2174 mg, nicotinamide 7000 mg, pyridoxine hydrochloride 700 mg, biotin 30 mg, folic acid 110 mg, cyanocobalamine 2 mg, calcium iodinate 108 mg, MgO 198,991 mg, MnSO_4_ 32,985 mg, ZnSO_4_ 19,753 mg, FeSO_4_ 43,523 mg, CuSO_4_ 4019 mg, and choline chloride 299,608 mg.

### 2.3 Analysis of Skeletal Muscle Protein Turnover

Rates of skeletal muscle protein degradation and synthesis were estimated using N^τ^‐MeHis content in the skeletal muscles and excretion of chicks were analyzed according to the methods of Hayashi et al. (1985). In brief, the samples of excreta were weighed into Erlenmeyer flasks and hydrolyzed with 6 mol/L hydrochloric acid in an autoclave (115°C) for 20 h. The hydrolysates were cooled and passed through filter paper; after hydrochloric acid was removed by evaporation under reduced pressure, the hydrolysates were dissolved in water and evaporated again in the presence of a small amount of sodium hydroxide to facilitate removal of ammonia. The residue was dissolved and the volume was adjusted to 25 mL with 0.2 mol/L pyridine. Subsequently, N^τ^‐MeHis was roughly separated from acidic and neutral amino acids by ion‐exchange chromatography, and its concentration was determined by high‐performance liquid chromatography according to the method previously described (Hayashi et al. 1985; Ohtsuka et al. 2011).

Before calculating protein turnover rates, the distribution of N^τ^‐MeHis among the tissues and organs in 5‐day‐old chicks was examined (Table S1). The tissue distribution of N^τ^‐MeHis in 5‐day‐old chicks in the present study was nearly identical to that reported for 15‐day‐old broilers by Hayashi et al. (1985). Skeletal muscle protein synthesis and degradation rates were estimated using the equations originally developed by Funabiki et al. (1976) and later applied by Hayashi et al. (1985). Following these studies, the calculations were performed under the following assumptions: (i) Approximately 80% of excreted N^τ^‐MeHis is derived from skeletal muscle, assuming that the contribution of nonskeletal muscle tissues to total N^τ^‐MeHis excretion in chicks is comparable to that reported in rats (Nishizawa et al. 1977), as adopted by Hayashi et al. (1985) in chickens, and (ii) all tissues containing N^τ^‐MeHis synthesize and degrade proteins at the same fractional rate as skeletal muscle.

Total N^τ^‐MeHis pool size in skeletal muscle was calculated using whole skeletal muscle weight and the N^τ^‐MeHis content of skeletal muscle. The daily excretion of N^τ^‐MeHis was calculated using the N^τ^‐MeHis content of excreta and the amount of excreta collected per day. Because the dietary N^τ^‐MeHis content was trace, N^τ^‐MeHis originating from the diet was excluded from the calculations. Furthermore, the skeletal‐muscle N^τ^‐MeHis pool size at Day 5 (P_0_) was estimated by subtracting the amount of N^τ^‐MeHis newly accumulated because of muscle growth between Days 5 and 7 from the total skeletal‐muscle N^τ^‐MeHis pool size at Day 7. The newly accumulated N^τ^‐MeHis was calculated using the body weight gain from Days 5 to 7, the relative skeletal muscle mass at Day 7, and the N^τ^‐MeHis content in skeletal muscle, all of which are summarized in Table 3. This estimation was based on the assumption that body weight gain during this period was allocated to skeletal muscle in proportion to the muscle‐to‐body‐weight ratio at Day 7.

**TABLE 3:** Muscle protein synthesis (Ks) and degradation (Kd) rates in 7‐day‐old fast‐ and slow‐growing chicks.

Body weight (g)	Slow‐growing	Fast‐growing	
At 1 day of age	40.88 ± 1.05	41.52 ± 0.80	
At 5 days of age	64.07 ± 1.07	94.88 ± 0.92	*
At 7 days of age	97.60 ± 2.80	126.99 ± 2.86	*
Body weight gain (g/day)	9.45 ± 0.40	14.25 ± 0.46	*
Feed intake (g/day)	18.85 ± 0.65	23.21 ± 0.50	*
Whole‐skeletal muscle weight (g)	20.55 ± 1.36	29.84 ± 1.48	*
Whole‐skeletal muscle (g/100 g body weight)	20.94 ± 0.83	23.43 ± 0.57	*
N^τ^‐MeHis content in skeletal muscle (μmol/g muscle)	0.41 ± 0.03	0.39 ± 0.01	
N^τ^‐MeHis excretion (μmol/day)	0.52 ± 0.06	0.44 ± 0.04	
Protein synthesis rate in skeletal muscle (Ks, %/d)	13.59 ± 0.36	14.33 ± 0.25	
Protein degradation in skeletal muscle (Kd, %/d)	4.27 ± 0.30	3.46 ± 0.21	*

*Note:* *p < 0.05 (by *t*‐test). Results are expressed as mean ± standard error of the mean (*n* = 6 cages).

### 2.4 Sample Preparation for GC‐MS/MS Analysis

Plasma supernatant (50 μL), along with pectoralis major muscle (20 mg) and liver (20 mg), was subjected to metabolomic analysis. The samples were treated in accordance with a modified version of a previously described method (Shigematsu et al. 2016; Shimamoto et al. 2020). They were suspended in 250 μL of methanol/chloroform/water (5:2:2), with 5 μL of 1 mg/mL 2‐isopropylmalic acid as the internal standard. The hydrophilic fraction was extracted and derivatized by methoxyamine hydrochloride and N‐methyl‐N‐(trimethylsilyl)trifluoroacetamide. To prepare trimethylsilyl derivatives, the tubes were shaken at 1200 × g and 37°C for 45 min in the dark.

GC/MS analysis was performed as previously described (Tomonaga et al. 2022), using a GC/MS‐QP2010SE (Shimadzu Corporation, Kyoto, Japan). As the GC column, a DB‐5 (30 m × 0.25 mm i.d., film thickness 1.00 μm; GL Science, Tokyo, Japan). The GC column temperature was programmed to maintain an initial temperature of 100°C for 4 min, then increased to 320°C at a rate of 10°C/min, and finally maintained at 320°C for 11 min, producing a total GC run time of 37 min. The inlet temperature was maintained at 280°C, and helium was used as the carrier gas at a constant flow rate of 39.0 cm/s. A sample of 1.0 μL was injected in splitless mode, and the mass conditions were as follows: ionization voltage, 70 eV; ion source temperature, 200°C; and full scan mode in the m/z range 45–600 at an interval of 0.3 s. The chromatogram acquisition and detection of mass spectral peaks and the processing of their waveforms were performed using Shimadzu GC/MS solution software (Shimadzu Corporation).

### 2.5 Data Processing for Untargeted Metabolomics

The samples were treated in accordance with a previously described method (Tomonaga et al. 2022). The GC‐MS analysis data were exported in net CDF format and then converted to ABF format, and peak detection and alignment were performed using MS‐DIAL version 4.92 (Lai et al. 2018). To minimize the number of missing values, peaks with similarity of > 70% and a retention index within ±10% were accepted by comparison with the Smart Metabolites Database (Shimadzu Corporation). Metabolite levels were semi‐quantified using the peak area of each metabolite relative to the internal standard (2‐isopropylmalic acid). The mean of each metabolite in the intermediate‐growing chickens was set to 100.

### 2.6 RNA Extraction and Quantitative Real‐Time PCR

The pectoralis major muscle and liver were homogenized in ISOGEN II (Nippon Gene, Tokyo, Japan), in accordance with the manufacturer's instructions. Real‐time PCR was performed as described previously (Shimamoto et al. 2016). In brief, cDNA was synthesized from 60 ng of RNA per 10 μL of reaction solution using the PrimeScript RT Reagent Kit (RR036A; Takara, Shiga, Japan). Samples were incubated at 37°C for 15 min, 85°C for 5 s, and 4°C for 5 min. Gene expression levels were measured by real‐time PCR using the StepOnePlus Real‐Time PCR System (Applied Biosystems, Foster City, CA, USA) with SYBR Select Master Mix (Applied Biosystems). Thermal cycling conditions were as follows: an initial hold at 50°C for 2 min, 95°C for 2 min, and then 45 cycles at 95°C for 15 s, 55°C for 15 s, and 72°C for 1 min. The primers used in this study are listed in Table 4. Each sample was run in duplicate along with no template and negative RT controls in each plate. Efficiencies and *R* ^2^ were assessed using five serial dilutions of cDNA: PCRs were highly specific and reproducible (*R* ^2^ = 0.98–1.00) and all primer pairs had equivalent PCR efficiency (from 98% to 105%). Melting curves were created and revealed a single peak for all primer pairs. Coefficients of variations were 7%–11%. Amplification, creation of dissociation curves, and gene expression analysis were performed using Dissociation Curves software (Applied Biosystems). Because there were no significant differences of cycle threshold values of 18S ribosomal RNA among each of the groups, the level of 18S ribosomal RNA was used as an internal standard. The results of gene expression levels are shown as a ratio to the value of chicks in the fast‐growing group.

**TABLE 4:** List of primer sequences used for quantitative real time polymerase chain reaction.

Gene		Sequence (5′‐3′)	Accession no.
*BCAT1*	Forward	CAC GAT GAA GGG CTG TCA GA	XM_416424.7
	Reverse	AGT CAC GTA CTT TGT TGC TTC C	
*BCKDH*	Forward	ACC TCT TCT CCG ATG TGT ACC G	XM_025144505.3
	Reverse	TCG TAG AGC TCC ATG GGG TAA T	
*18S ribosomal RNA*	Forward	AAA CGG CTA CCA CAT CCA AG	XR_006936393.1
	Reverse	CCT CCA ATG GAT CCT CGT TA	

Abbreviations: BCAT1, branched‐chain aminotransferase 1; BCKDH, branched‐chain α‐keto acid dehydrogenase.

### 2.7 Statistical Analysis

The data were analyzed using one‐way ANOVA, with individual comparisons being made using Tukey's post hoc test. A *t*‐test was used to compare the mean values of the slow‐growing and fast‐growing groups. The analysis was performed using R (R Development Core Team 2025). For all comparisons, statistical significance was set at *p* < 0.05. The metabolomics results were used to discriminate sample patterns by partial least squares discriminant analysis (PLS‐DA), using MetaboAnalyst software Version 6.0 (Pang et al. 2024). In addition, quantitative enrichment analysis using pathway‐related metabolite sets included in MetaboAnalyst 6.0, as an established tool for metabolite set enrichment analysis, was performed with metabolite compounds from plasma, muscle, and liver that significantly differed between the groups as determined by one‐way ANOVA, with differences considered significant at *p* < 0.05.

## 3 Results and Discussion

In experiment 1, the slow‐ and fast groups of chicks were selected based on their body weight gain up to 5 days of age. The difference in their body weight was maintained until 7 days of age (Table 3). Previous studies reported that the rate of muscle protein degradation may make a greater contribution to body growth of chickens than the rate of muscle protein synthesis (Hayashi et al. 1985; Maeda et al. 1987; Tomas et al. 1988). According to the methods of the previous studies (Funabiki et al. 1976; Nishizawa et al. 1977; Hayashi et al. 1985), we determined the total N^τ^‐MeHis content of whole‐body skeletal muscle and its daily excretion of the fast‐ and slow‐growing groups of chicks to estimate the muscle protein turnover rate. We found that the rate of muscle protein degradation was significantly lower in the fast‐growing group than in the slow‐growing group, while the rates of muscle protein synthesis did not differ between them (Table 3). These results suggest that protein degradation levels determine individual differences in body weight gain of broiler chicks during the neonatal period.

In experiment 2, the slow‐, intermediate, and fast groups of chicks were selected based on their body weight gain up to 5 days of age. Consequently, the body weight at 5 days of age was significantly different among the groups (60.43 ± 0.81, 76.02 ± 0.63, and 89.86 ± 1.88 g), despite the fact that initial body weight did not differ among them (40.83 ± 0.70, 41.99 ± 0.72, and 41.36 ± 1.12 g). The pectoralis major muscle, thigh muscle, and liver in the fast‐growing group were heavier than those in the slow‐growing group (Table 5). In particular, the ratio of pectoralis muscle weight to body weight at 5 days of age was higher in the fast‐growing chicks than in the slow‐growing ones, while the ratio of thigh muscle weight to body weight did not differ significantly among the three groups (Table 5). Meanwhile, the ratio of liver weight to body weight was lower in the fast‐growing chicks than in the slow‐growing ones (Table 5). These results are in agreement with the results of our previous study (Shimamoto et al. 2018) and the work of Brown et al. (1985), who reported that the ratio of liver weight to body weight is inversely correlated with the body weight of chickens. These results suggested that the pectoralis muscle of the fast‐growing chicks was enlarged until 5 days of age compared with that of their slow‐growing counterparts.

**TABLE 5:** The body weight and tissue weights in the slow‐, intermediate‐, and fast‐growing chicks.

	Slow‐growing	Intermediate‐growing	Fast‐growing
Initial body weight (g)	40.83 ± 0.70	41.99 ± 0.72	41.36 ± 1.12
Final body weight (g)	60.43 ± 0.81c	76.02 ± 0.63b	89.86 ± 1.88a
Pectoralis major muscle weight (g)	1.40 ± 0.09c	2.89 ± 0.14b	3.91 ± 0.18a
Thigh weight (g)	9.41 ± 0.40c	11.48 ± 0.27b	13.39 ± 0.36a
Liver weight (g)	2.27 ± 0.07b	2.71 ± 0.02a	2.91 ± 0.08a
Pectoralis major muscle (g/100 g body weight)	2.31 ± 0.14b	3.80 ± 0.19a	4.34 ± 0.12a
Thigh (g/100 g body weight)	15.55 ± 0.59	15.09 ± 0.29	14.92 ± 0.39
Liver (g/100 g body weight)	3.77 ± 0.12b	3.56 ± 0.05ab	3.24 ± 0.09a

*Note:* Different letters in the same row indicate significant differences by Tukey's post hoc test (*p* < 0.05). Results are expressed as mean ± standard error of the mean (*n* = 8).

**FIGURE 1:**
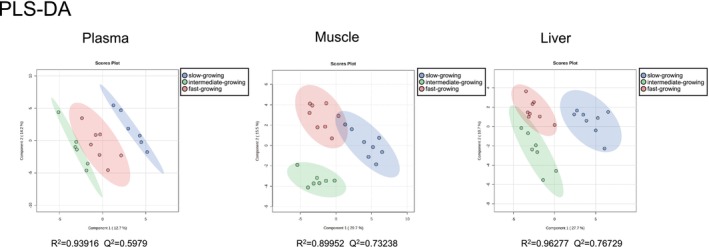
PLS‐DA score plots of metabolite data in plasma, muscle, and liver of chicks with different rates of body growth. Slow‐growing chicks are shown as blue, intermediate‐growing chicks are shown as green, and fast‐growing chicks are shown as red. The sample size ranged from 6 to 8 for each group and each tissue.

Next, to investigate the reasons for the differences in muscle protein degradation rate and body weight gain, we performed GC‐MS–based untargeted metabolomics and compared the relative amounts of metabolites in plasma, muscle (pectoralis major muscle), and liver among the three groups with different growth rates. This analysis identified a total of 59, 100, and 102 metabolites in plasma, muscle, and liver, respectively (Table S2). PLS‐DA of all metabolites revealed that their clustering that did not align with the three groups of chicks; in other words, the metabolite profiles differed among these groups in plasma, muscle, and liver (Figure 1).

By one‐way ANOVA, we confirmed that the levels of 9, 16, and 23 metabolites were significantly different in plasma, muscle, and liver, respectively. Among them, we found that 4, 14, and 22 metabolites were correlated with the body weight gain of chicks from 1 to 5 days of age when Pearson's correlation coefficient threshold was set to |*r*| ≥ 0.4 (Table 6). To obtain information about the metabolic pathways associated with the protein degradation rate, we performed pathway analysis by using metabolites shown in Table 7 and found that four, five, and eight pathways were particularly associated with the plasma, muscle, and liver, respectively (Table 7). Most of the metabolic pathways associated with plasma and muscle were involved in protein and amino acid metabolism, while those in liver were related to the tricarboxylic acid (TCA) cycle and carbohydrate metabolism.

**TABLE 6:** Metabolite levels in plasma, muscle, and liver correlate with in the slow‐, intermediate‐, and fast‐growing chicks.

Plasma	Slow‐growing	Intermediate‐growing	Fast‐growing	SEM	Pearson's correlation
2‐Hydroxyisobutyric acid	104.87	100.00	57.95	6.50	−0.64
Valine	118.11	100.00	199.94	14.26	0.48
Leucine	117.86	100.00	320.52	32.62	0.50
2‐Aminobutyric acid	95.26	100.00	204.81	17.50	0.54
Muscle					
Ribose	158.90	100.00	63.74	12.13	−0.70
Maltose	249.87	100.00	68.28	25.08	−0.67
Serine	83.12	100.00	36.48	5.86	−0.62
Phosphoric acid	129.98	100.00	63.10	10.56	−0.59
Mannitol	270.08	100.00	89.88	31.70	−0.55
Citric acid	30.78	100.00	78.35	10.53	0.40
4‐Hydroxyproline	51.72	100.00	80.54	6.95	0.44
Leucine	70.55	100.00	135.91	10.71	0.44
Malonic acid	72.72	100.00	102.22	5.60	0.47
3‐Hydroxyisobutyric acid	105.82	100.00	150.18	7.12	0.53
Valine	82.02	100.00	127.95	6.67	0.54
Proline	73.18	100.00	108.13	5.79	0.54
Phenylalanine	44.77	100.00	123.71	11.86	0.57
2‐Aminobutyric acid	47.96	100.00	105.82	7.30	0.68
Liver					
Sorbose	185.75	100.00	69.86	13.27	−0.75
Fumaric acid	252.79	100.00	53.62	25.20	−0.69
Ribulose	159.51	100.00	66.50	11.99	−0.67
Lactose	316.10	100.00	67.44	31.95	−0.67
Arabinose	127.04	100.00	76.95	6.80	−0.63
4‐Hydroxyphenylacetic acid	184.69	100.00	49.60	17.91	−0.62
Malic acid	206.83	100.00	53.44	21.27	−0.62
Methylmalonic acid	214.82	100.00	72.88	20.02	−0.60
Glycerol	125.33	100.00	82.88	5.99	−0.58
Serine	141.18	100.00	69.26	11.16	−0.57
Fructose 1‐phosphate	338.50	100.00	61.96	41.82	−0.57
5‐Aminovaleric acid	299.12	100.00	99.35	34.13	−0.55
2‐Hydroxyglutaric acid	114.65	100.00	68.11	7.22	−0.55
Galactose	118.91	100.00	47.08	11.39	−0.51
1,5‐Anhydro‐glucitol	105.82	100.00	74.44	5.24	−0.50
Threonine	161.01	100.00	118.63	9.44	−0.45
2‐Hydroxybutyric acid	95.78	100.00	137.29	7.65	0.40
Galactitol	70.73	100.00	192.31	19.19	0.46
2‐Phosphoglyceric acid	38.35	100.00	97.97	9.89	0.50
Fructose 6‐phosphate	385.61	100.00	50.07	51.45	0.56
Boric acid	75.96	100.00	136.02	7.91	0.63
2‐Aminobutyric acid	54.37	100.00	195.20	17.31	0.66

*Note:* Slow‐growing, intermediate‐growing, and fast‐growing groups reflect growth rates at 1–5 days of age. The results are shown for *p* < 0.05 by one‐way analysis of variance and |*r*| ≥ 0.4 by Pearson's correlation analysis. The units are relative values. *n* = 6–8 in each group.

**TABLE 7:** Potential metabolic pathways affected by protein degradation rate.

Metabolism name	*p*		
	Plasma	Muscle	Liver
Glycine, serine, and threonine metabolism	*		*
Cysteine and methionine metabolism	*		*
Arginine and proline metabolism	*	*	
Valine, leucine, and isoleucine degradation	*	*	
Ammonia recycling		*	
Aspartate metabolism		*	
Transfer of acetyl groups into mitochondria		*	
Galactose metabolism			*
Pentose and glucuronate interconversions			*
Citrate cycle (TCA cycle)			*
Fructose and mannose metabolism			*
Pyruvate metabolism			*
Glyoxylate and dicarboxylate metabolism			*

*p < 0.05.

Intriguingly, because valine and leucine (both of which are branched‐chain amino acids [BCAAs]) in plasma and skeletal muscle showed positive correlations with body weight gain, we focused on the BCAA metabolism of the chicks. A rise in the intracellular concentration of BCAAs is known to upregulate protein synthesis and downregulate protein degradation in skeletal muscles (May and Buse 1989; Nakashima et al. 2005). The anabolic effects of the BCAAs, especially leucine, are mediated in part through the activation of the mammalian/mechanistic target of rapamycin complex 1 (mTORC1). It has also been reported that mTORC1 inhibits muscle protein degradation by activating either the ubiquitin proteasome pathway or the autophagy/lysosomal pathways (Bodine et al. 2001; Nakashima and Ishida 2020). These results suggested that intracellular leucine concentration might contribute to the difference in muscle protein degradation rate of the chicks. Furthermore, in our previous study, we reported that the mRNA expression of muscle atrophy F‐box (atrogin‐1/MAFbx), which is a muscle‐specific ubiquitin ligase, was higher in slow‐growing chicks than in fast‐growing ones at 5 days of age (Shimamoto et al. 2018). This raises the possibility that the intracellular leucine concentration is related to that via regulating the ubiquitin proteasome pathway in the skeletal muscle of broiler chicks.

**FIGURE 2:**
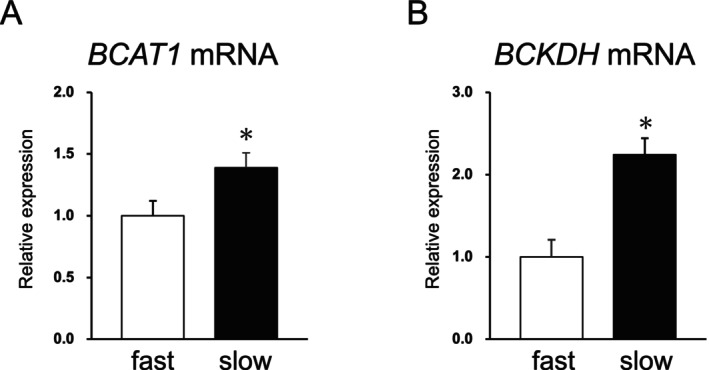
Gene expression in the pectoralis muscle or liver in 5‐day‐old chicks. Expression of *BCAT1* mRNA in the pectoralis muscle (A) and *BCKDH* mRNA in the liver (B). Results are expressed as mean ± standard error of the mean (*n* = 8). *p < 0.05. BCAT, branched‐chain aminotransferase; BCKDH, branched‐chain α‐keto acid dehydrogenase complex; fast, the fast‐growing chick group; slow, the slow‐growing chick group.

Meanwhile, although the reasons for the lower concentrations of valine and leucine in the slow‐growing group remain unclear, one possible explanation is the difference in their degradation pathway compared with that of other amino acids. BCAAs are metabolized and used as energy sources (Harper et al. 1984). The first two steps of BCAA catabolism occur in muscle and liver; the first step is transamination catalyzed by branched‐chain aminotransferase (BCAT), while the second step is oxidative decarboxylation catalyzed by the branched‐chain α‐keto acid dehydrogenase complex (BCKDH) (Harper et al. 1984). Interestingly, the expression levels of BCAT are high in the skeletal muscle and low in the liver (Harper et al. 1984). Meanwhile, the second BCKDH reaction is known to be the rate‐limiting step in BCAA catabolism (Harper et al. 1984; Harris et al. 1990), and this enzyme exhibits its peak activity in the liver (Harper et al. 1984). In this study, we found that, in muscle, *BCAT1* mRNA expression was significantly higher in the slow‐growing chicks than in their fast‐growing counterparts at 5 days of age (Figure 2A). In addition, in the liver, *BCKDH* mRNA expression was higher in the slow‐growing chicks than in the fast‐growing ones (Figure 2B). These results suggested that, because the activity of BCAA degradation was high, the concentrations of valine and leucine were low in the slow‐growing chicks, and consequently a higher rate of muscle protein degradation rate might have been maintained compared with that in their fast‐growing counterparts.

Furthermore, it has been reported that isovaleryl‐CoA derived from leucine is metabolized to acetyl‐CoA, while isobutyryl‐CoA derived from valine is metabolized to propionyl‐CoA, which can be used in the TCA cycle (Adeva‐Andany et al. 2017). Our GC‐MS–based metabolomic analysis indicated that the levels of intermediate metabolites of the TCA cycle (i.e., fumaric acid and malic acid) were higher in the liver of the slow‐growing group than in fast‐growing chicks. In addition, the level of fructose 6‐phosphate, an intermediate metabolite of glycolysis, was also higher in the liver of the slow‐growing group than in the fast‐growing chicks. Furthermore, in the liver, multiple sugars and their metabolites were suggested to show higher levels in the livers of slow‐growing chicks than in fast‐growing ones. These results suggested that, in the slow‐growing chicks, amino acids including valine and leucine were degraded and used as energy sources, unlike in their fast‐growing counterparts.

Our GC‐MS–based metabolomics also indicated that serine and threonine were negatively correlated with the body weight gain in muscle (serine) and liver (serine and threonine), respectively. Serine and threonine are known to be precursors of glycine, an amino acid that is essential for growth in chickens, which is involved in the synthesis of collagen, heme, glutathione, and creatine (Kidd and Kerr 1996; Stevens 1996; Shoulders and Raines 2009). Especially in broiler chickens, a deficiency of glycine markedly inhibited their growth (Wang et al. 2013). Moreover, serine is interconverted to glycine in the presence of serine hydroxymethyltransferase and threonine is degraded to glycine by threonine dehydrogenase and threonine aldolase (Baker and Sugahara 1970). It has been reported that broilers fed a low‐protein diet exhibit an elevated rate of body protein degradation, as evidenced by increased N^τ^‐MeHis release levels (Hocking and Saunderson 1992). Fancher and Jensen (1989) observed that broilers fed a low‐protein diet showed increased plasma threonine levels. In addition, such a diet inhibited liver threonine dehydrogenase activity, leading to reduced glycine synthesis (Davis and Austic 1997). These results suggested that chicks with high protein degradation rates, such as slow‐growing chicks, may not need to catabolize threonine or serine into glycine because they obtain sufficient glycine from the degradation of body proteins.

Furthermore, among the metabolites, 2‐aminobutyric acid, produced as a result of threonine catabolism, in plasma, muscle, and liver exhibited positive correlations with the body weight gain. These results suggest that threonine catabolic activity was lower in the slow‐growing chicks than in the fast‐growing ones. In addition, 2‐aminobutyric acid is also biosynthesized as a byproduct of the synthesis of cysteine. Irino et al. (2016) reported that 2‐aminobutyric acid increases intracellular glutathione levels in myocardium and exerts protective effects against oxidative stress. Furthermore, it has been shown that mitochondrial superoxide production enhances UPS‐dependent proteolysis via the production of *atrogin‐1/MAFbx* mRNA in cultured chick myotube (Furukawa et al. 2015). In this study, because 2‐aminobutyric acid was positively correlated with body weight gain, oxidative stress levels might be negatively correlated with either muscle protein degradation levels or the body weight gain in broiler chicks during the neonatal period.

It should be noted that the sampling ages, rearing environments, and diet compositions were not entirely consistent between the experiment 1 (protein degradation rate measurement) and the experiment 2 (metabolomic analysis). Furthermore, differences in feed intake may have influenced the muscle protein degradation rate. In fact, in the experiment 1, feed intake was higher in the fast‐growing group than in the slow‐growing group (Table 3). These variations in rearing environment, diet composition, and feed intake could have affected the observed metabolite profiles and gene expression. In this study, based on the results of Experiment 1, we focused on the significant difference in protein degradation rates between the fast‐ and slow‐growing groups. However, the difference in protein synthesis rates between the two groups was also comparable to that observed for degradation rates. Saunderson and Leslie (1988) compared broilers (fast‐growing) with layers (slow‐growing) and demonstrated that rapidly growing broilers tend to have lower rates of protein degradation. In addition, they suggested that differences in protein synthesis rates also contribute substantially to muscle growth, particularly in chicks younger than 2 weeks of age. Therefore, a more comprehensive understanding of the metabolic characteristics of fast‐growing chicks, particularly their combination of accelerated protein synthesis and reduced protein degradation, may provide important insights for breeding strategies aimed at improving growth efficiency.

In summary, GC‐MS–based metabolomics revealed that 4, 14, and 22 metabolites from plasma, muscle, and liver, respectively, were correlated with the body weight gain of chicks from 1 to 5 days of age. Most of them were involved in either amino acid metabolism or carbohydrate metabolism. In addition, because the slow‐growing chicks with a higher rate of muscle protein degradation showed higher mRNA expression of enzymes related to BCAA catabolism, BCAA catabolism might be important for the rate of muscle protein degradation of broiler chicks.

## Author Contributions

Saki Shimamoto, Hanae Katayama, Daichi Ijiri, Shinobu Fujimura, Kazuki Nakashima, Shinya Ishihara, and Akira Ohtsuka conceived and designed the experiments. Saki Shimamoto and Hanae Katayama performed all of the experiments, and Shinya Ishihara, Shozo Tomonaga, and Miyu Kamimura contributed reagents/materials/analysis tools. Saki Shimamoto and Daichi Ijiri wrote the paper. Saki Shimamoto, Daichi Ijiri, Shinobu Fujimura, Kazuki Nakashima, and Akira Ohtsuka reviewed and edited the paper. Saki Shimamoto administered the project. All authors have read and agreed to the published version of the manuscript.

## Funding

This work was supported by the Japan Society for the Promotion of Science (21K14958).

## Conflicts of Interest

The authors declare no conflicts of interest.

## Supporting information


**Table S1:** Distribution of N^τ^‐methylhistidine among organs and tissues in 5‐day‐old chicks.
**Table S2:** Metabolite levels in plasma, muscle, and liver with in the slow‐, intermediate‐, and fast‐growing chicks.
