# Supplementary material for: Metabolomic Profiles Associated With Difference in Muscle Protein Degradation Between Fast‐ and Slow‐Growing Chicks During the Neonatal Period
Source: Anim Sci J. 2026 Mar 31;97(1):e70169. doi: 10.1111/asj.70169 (PMC13037696; doi:10.1111/asj.70169)
Supplement: Supplementary file 1 — Table S1: Distribution of Nτ‐methylhistidine among organs and tissues in 5‐day‐old chicks. Table S2: Metabolite levels in plasma, muscle, and liver with in the slow‐, intermediate‐, and fast‐growing chicks. [file ASJ-97-e70169-s001.docx]

| Table S1. Distribution of N^τ^-Methylhistidine among organs and tissues in 5-day-old chicks. | | | | | | | | | | |
| --- | --- | --- | --- | --- | --- | --- | --- | --- | --- | --- |
|  |  |  |  |  | N^τ^-Methylhistidine | | | | |  |
|  | Fresh weight (g) | | |  | (μmol) | | |  |  |  |
|  | Mean |  | SE |  | Mean |  | SE |  | (%) |  |
| Skeletal muscle | 16.88 | ± | 0.91 |  | 8.1 | ± | 0.61 |  | 64.4 |  |
| Stomach | 3.44 | ± | 0.23 |  | 1.4 | ± | 0.29 |  | 14.2 |  |
| Heart | 0.66 | ± | 0.04 |  | 0.2 | ± | 0.01 |  | 1.1 |  |
| Liver | 2.92 | ± | 0.11 |  | 0.2 | ± | 0.03 |  | 1.0 |  |
| Skin and feather | 7.34 | ± | 0.20 |  | 0.5 | ± | 0.12 |  | 3.6 |  |
| Blood, bone, and head | 22.99 | ± | 0.64 |  | 1.1 | ± | 0.29 |  | 7.0 |  |
| Others | 13.45 | ± | 0.57 |  | 0.7 | ± | 0.14 |  | 8.8 |  |
| Results are expressed as mean ± standard error of the mean (n=6). | | | | | | | | | | |

| Table S2. Metabolite levels in plasma, muscle, and liver with in the slow-, intermediate-, and fast-growing chicks. | | | | | |  | |  | |
| --- | --- | --- | --- | --- | --- | --- | --- | --- | --- |
| Plasma | slow-growing | intermediate-growing | fast-growing | SEM |  | |  | |  |
| 1,5-Anhydro-glucitol | 77.65 | 100.00 | 44.47 | 8.21 |  | |  | |  |
| 2-Aminobutyric acid | 95.26 | 100.00 | 204.81 | 17.89 |  | |  | |  |
| 2-Deoxytetronic acid | 94.34 | 100.00 | 119.71 | 6.68 |  | |  | |  |
| 2-Hydroxyisobutyric acid | 104.87 | 100.00 | 57.95 | 6.65 |  | |  | |  |
| 2-Ketoglutaric acid | 65.85 | 100.00 | 62.51 | 8.87 |  | |  | |  |
| 2-Ketoisocaproic acid | 87.55 | 100.00 | 111.40 | 5.93 |  | |  | |  |
| 3-Aminopropanoic acid | 200.11 | 100.00 | 242.31 | 28.42 |  | |  | |  |
| 3-Hydroxybutyric acid | 72.17 | 100.00 | 92.56 | 5.84 |  | |  | |  |
| 3-Hydroxypropionic acid | 85.12 | 100.00 | 76.11 | 7.34 |  | |  | |  |
| 4-Hydroxyphenylacetic acid | 165.49 | 100.00 | 126.88 | 11.20 |  | |  | |  |
| 4-Hydroxyproline | 248.41 | 100.00 | 281.73 | 39.43 |  | |  | |  |
| 5-Aminolevulinic acid | 392.12 | 100.00 | 779.45 | 97.55 |  | |  | |  |
| Alanine | 113.19 | 100.00 | 192.11 | 20.38 |  | |  | |  |
| Arabinose | 78.22 | 100.00 | 81.45 | 10.36 |  | |  | |  |
| Arabitol | 92.06 | 100.00 | 97.61 | 5.92 |  | |  | |  |
| Benzoic acid | 99.60 | 100.00 | 103.23 | 2.52 |  | |  | |  |
| Cholesterol | 107.45 | 100.00 | 74.71 | 7.84 |  | |  | |  |
| Citraconic acid | 114.51 | 100.00 | 114.65 | 9.20 |  | |  | |  |
| Citric acid | 115.47 | 100.00 | 156.84 | 20.17 |  | |  | |  |
| Dimethylglycine | 142.06 | 100.00 | 117.00 | 7.27 |  | |  | |  |
| Ethylhydracrylic acid | 109.00 | 100.00 | 126.60 | 7.71 |  | |  | |  |
| Fructose | 215.25 | 100.00 | 155.84 | 25.16 |  | |  | |  |
| Fucose | 96.39 | 100.00 | 95.67 | 2.33 |  | |  | |  |
| Glucose | 93.57 | 100.00 | 92.15 | 3.37 |  | |  | |  |
| Glucuronic acid | 150.73 | 100.00 | 101.28 | 11.07 |  | |  | |  |
| Glyceric acid | 90.61 | 100.00 | 103.30 | 4.83 |  | |  | |  |
| Glycerol | 150.62 | 100.00 | 175.64 | 18.54 |  | |  | |  |
| Glycine | 147.65 | 100.00 | 157.25 | 17.37 |  | |  | |  |
| Glycolic acid | 102.22 | 100.00 | 95.44 | 4.18 |  | |  | |  |
| Inositol | 113.40 | 100.00 | 94.73 | 6.27 |  | |  | |  |
| Lactic acid | 95.92 | 100.00 | 107.73 | 6.21 |  | |  | |  |
| Leucine | 117.86 | 100.00 | 320.52 | 33.35 |  | |  | |  |
| Linoleic acid | 114.42 | 100.00 | 115.63 | 8.48 |  | |  | |  |
| Malic acid | 96.38 | 100.00 | 103.70 | 5.99 |  | |  | |  |
| Mannitol | 236.08 | 100.00 | 129.20 | 22.21 |  | |  | |  |
| Mannose | 84.83 | 100.00 | 73.93 | 5.41 |  | |  | |  |
| meso-Erythritol | 113.58 | 100.00 | 99.88 | 4.20 |  | |  | |  |
| Monostearin | 79.90 | 100.00 | 60.56 | 5.79 |  | |  | |  |
| Myristic acid | 106.47 | 100.00 | 109.43 | 6.51 |  | |  | |  |
| Nonanoic acid | 101.50 | 100.00 | 99.70 | 5.36 |  | |  | |  |
| Oleic acid | 92.39 | 100.00 | 103.62 | 9.67 |  | |  | |  |
| Palmitic acid | 96.41 | 100.00 | 99.69 | 5.96 |  | |  | |  |
| Phosphoric acid | 121.53 | 100.00 | 163.39 | 15.42 |  | |  | |  |
| Proline | 171.23 | 100.00 | 293.82 | 31.57 |  | |  | |  |
| Pyruvic acid | 105.79 | 100.00 | 97.20 | 5.82 |  | |  | |  |
| Ribitol | 83.56 | 100.00 | 90.69 | 3.69 |  | |  | |  |
| Ribose | 78.79 | 100.00 | 83.22 | 10.46 |  | |  | |  |
| Ribulose | 87.11 | 100.00 | 96.93 | 6.06 |  | |  | |  |
| Serine | 169.58 | 100.00 | 114.44 | 19.02 |  | |  | |  |
| Sorbose | 129.93 | 100.00 | 140.55 | 7.95 |  | |  | |  |
| Stearic acid | 118.15 | 100.00 | 124.38 | 11.08 |  | |  | |  |
| Succinic acid | 220.86 | 100.00 | 233.08 | 29.51 |  | |  | |  |
| Sucrose | 129.98 | 100.00 | 188.27 | 22.61 |  | |  | |  |
| Threitol | 106.25 | 100.00 | 94.67 | 3.42 |  | |  | |  |
| Threonic acid | 82.58 | 100.00 | 103.31 | 4.63 |  | |  | |  |
| Threonine | 283.43 | 100.00 | 208.16 | 24.53 |  | |  | |  |
| Urea | 169.95 | 100.00 | 201.24 | 25.67 |  | |  | |  |
| Uridine | 39.20 | 100.00 | 144.23 | 16.43 |  | |  | |  |
| Valine | 118.11 | 100.00 | 199.94 | 14.58 |  | |  | |  |
| Muscle |  |  |  |  |  | |  | |  |
| 1,5-Anhydro-glucitol | 139.85 | 100.00 | 98.81 | 9.59 |  | |  | |  |
| 1,6-Anhydroglucose | 127.00 | 100.00 | 78.67 | 10.68 |  | |  | |  |
| 2-Aminobutyric acid | 47.96 | 100.00 | 105.82 | 7.47 |  | |  | |  |
| 2-Hydroxybutyric acid | 83.28 | 100.00 | 108.87 | 5.06 |  | |  | |  |
| 2-Hydroxyglutaric acid | 97.59 | 100.00 | 82.60 | 5.56 |  | |  | |  |
| 2-Keto-isovaleric acid | 94.42 | 100.00 | 96.76 | 4.13 |  | |  | |  |
| 2-Ketobutyric acid | 168.07 | 100.00 | 85.89 | 18.64 |  | |  | |  |
| 2-Ketoglutaric acid | 106.76 | 100.00 | 61.15 | 11.49 |  | |  | |  |
| 2-Ketoisocaproic acid | 82.27 | 100.00 | 72.24 | 5.22 |  | |  | |  |
| 2-Methyl-3-hydroxyvaleric acid | 156.43 | 100.00 | 153.62 | 16.25 |  | |  | |  |
| 2-Phosphoglyceric acid | 144.01 | 100.00 | 105.41 | 14.09 |  | |  | |  |
| 3-Aminopropanoic acid | 53.39 | 100.00 | 58.04 | 11.84 |  | |  | |  |
| 3-Hydroxybutyric acid | 89.47 | 100.00 | 114.25 | 8.73 |  | |  | |  |
| 3-Hydroxyisobutyric acid | 105.82 | 100.00 | 150.18 | 7.28 |  | |  | |  |
| 3-Hydroxyisovaleric acid | 123.85 | 100.00 | 107.10 | 5.73 |  | |  | |  |
| 3-Hydroxypropionic acid | 98.17 | 100.00 | 105.04 | 2.32 |  | |  | |  |
| 3-Phosphoglyceric acid | 164.51 | 100.00 | 129.20 | 19.48 |  | |  | |  |
| 4-Aminobutyric acid | 124.92 | 100.00 | 85.86 | 8.24 |  | |  | |  |
| 4-Hydroxybutyric acid | 105.55 | 100.00 | 92.15 | 10.22 |  | |  | |  |
| 4-Hydroxyproline | 51.72 | 100.00 | 80.54 | 7.11 |  | |  | |  |
| 5-Aminovaleric acid | 103.95 | 100.00 | 102.88 | 8.75 |  | |  | |  |
| 5-Oxoproline | 123.37 | 100.00 | 97.02 | 5.38 |  | |  | |  |
| 5'-Methylthioadenosine | 68.37 | 100.00 | 95.99 | 7.03 |  | |  | |  |
| Alanine | 92.26 | 100.00 | 91.33 | 3.03 |  | |  | |  |
| Arabinose | 82.31 | 100.00 | 68.30 | 13.32 |  | |  | |  |
| Arabitol | 84.63 | 100.00 | 83.88 | 3.02 |  | |  | |  |
| Ascorbic acid | 44.49 | 100.00 | 58.10 | 7.81 |  | |  | |  |
| Aspartic acid | 92.51 | 100.00 | 80.13 | 10.99 |  | |  | |  |
| Azelaic acid | 97.75 | 100.00 | 78.84 | 10.97 |  | |  | |  |
| Boric acid | 81.01 | 100.00 | 89.17 | 4.92 |  | |  | |  |
| Citramalic acid | 82.97 | 100.00 | 68.78 | 5.94 |  | |  | |  |
| Citric acid | 30.78 | 100.00 | 78.35 | 10.76 |  | |  | |  |
| Creatinine | 91.23 | 100.00 | 90.94 | 2.32 |  | |  | |  |
| Cysteine | 65.46 | 100.00 | 103.49 | 8.40 |  | |  | |  |
| Dihydroxyacetone phosphate | 1115.19 | 100.00 | 696.60 | 198.95 |  | |  | |  |
| Dimethylglycine | 94.95 | 100.00 | 89.12 | 6.77 |  | |  | |  |
| Fructose 6-phosphate | 126.97 | 100.00 | 101.27 | 12.85 |  | |  | |  |
| Fructose | 127.09 | 100.00 | 77.42 | 8.98 |  | |  | |  |
| Fumaric acid | 119.28 | 100.00 | 89.20 | 6.04 |  | |  | |  |
| Galactitol | 97.64 | 100.00 | 84.90 | 4.09 |  | |  | |  |
| Galactose | 74.22 | 100.00 | 63.57 | 7.62 |  | |  | |  |
| Galacturonic acid | 96.63 | 100.00 | 83.09 | 6.69 |  | |  | |  |
| Gluconic acid | 63.96 | 100.00 | 76.48 | 7.07 |  | |  | |  |
| Glucose 6-phosphate | 131.45 | 100.00 | 104.87 | 14.09 |  | |  | |  |
| Glucose | 126.35 | 100.00 | 99.30 | 5.71 |  | |  | |  |
| Glucuronic acid | 157.51 | 100.00 | 102.01 | 12.32 |  | |  | |  |
| Glutamic acid | 56.40 | 100.00 | 101.60 | 10.49 |  | |  | |  |
| Glyceric acid | 130.98 | 100.00 | 108.42 | 10.82 |  | |  | |  |
| Glycerol 2-phosphate | 103.51 | 100.00 | 138.67 | 12.02 |  | |  | |  |
| Glycerol 3-phosphate | 91.29 | 100.00 | 151.31 | 16.94 |  | |  | |  |
| Glycerol | 107.10 | 100.00 | 95.33 | 2.48 |  | |  | |  |
| Glycine | 103.12 | 100.00 | 89.09 | 3.95 |  | |  | |  |
| Glycolic acid | 95.00 | 100.00 | 94.44 | 4.36 |  | |  | |  |
| Hypotaurine | 74.17 | 100.00 | 115.00 | 7.86 |  | |  | |  |
| Inositol | 102.79 | 100.00 | 94.33 | 1.85 |  | |  | |  |
| Isoleucine | 135.09 | 100.00 | 87.16 | 12.99 |  | |  | |  |
| Isomaltose | 108.23 | 100.00 | 92.62 | 10.75 |  | |  | |  |
| Lactic acid | 136.03 | 100.00 | 98.05 | 8.04 |  | |  | |  |
| Lactose | 83.23 | 100.00 | 39.49 | 12.04 |  | |  | |  |
| Lauric acid | 101.38 | 100.00 | 106.62 | 3.17 |  | |  | |  |
| Leucine | 70.55 | 100.00 | 135.91 | 10.95 |  | |  | |  |
| Malic acid | 99.61 | 100.00 | 92.97 | 4.47 |  | |  | |  |
| Malonic acid | 72.72 | 100.00 | 102.22 | 5.73 |  | |  | |  |
| Maltose | 249.87 | 100.00 | 68.28 | 25.64 |  | |  | |  |
| Mannitol | 270.08 | 100.00 | 89.88 | 32.41 |  | |  | |  |
| Mannose 6-phosphate | 129.48 | 100.00 | 101.07 | 13.33 |  | |  | |  |
| meso-Erythritol | 77.27 | 100.00 | 97.25 | 5.05 |  | |  | |  |
| Methylmalonic acid | 89.68 | 100.00 | 93.79 | 3.20 |  | |  | |  |
| Monostearin | 93.63 | 100.00 | 94.38 | 1.99 |  | |  | |  |
| Myristic acid | 101.38 | 100.00 | 97.95 | 1.48 |  | |  | |  |
| N-Acetylglutamine | 105.78 | 100.00 | 95.58 | 3.68 |  | |  | |  |
| N-Acetylmannosamine | 120.48 | 100.00 | 83.91 | 6.94 |  | |  | |  |
| Nonanoic acid | 110.30 | 100.00 | 88.38 | 4.00 |  | |  | |  |
| O-Phosphoethanolamine | 72.20 | 100.00 | 79.09 | 7.40 |  | |  | |  |
| Oxalic acid | 88.59 | 100.00 | 89.22 | 8.30 |  | |  | |  |
| Palmitic acid | 93.53 | 100.00 | 101.02 | 2.00 |  | |  | |  |
| Pantothenic acid | 73.02 | 100.00 | 83.70 | 6.22 |  | |  | |  |
| Phenylalanine | 44.77 | 100.00 | 123.71 | 12.12 |  | |  | |  |
| Phosphoenolpyruvic acid | 157.23 | 100.00 | 113.11 | 15.00 |  | |  | |  |
| Phosphoric acid | 129.98 | 100.00 | 63.10 | 10.79 |  | |  | |  |
| Proline | 73.18 | 100.00 | 108.13 | 5.92 |  | |  | |  |
| Putrescine | 71.38 | 100.00 | 105.82 | 8.29 |  | |  | |  |
| Pyruvic acid | 138.57 | 100.00 | 99.53 | 16.67 |  | |  | |  |
| Ribitol | 101.44 | 100.00 | 87.67 | 5.14 |  | |  | |  |
| Ribonic acid | 45.87 | 100.00 | 61.80 | 11.85 |  | |  | |  |
| Ribose | 158.90 | 100.00 | 63.74 | 12.40 |  | |  | |  |
| Ribulose 5-phosphate | 115.80 | 100.00 | 86.86 | 12.22 |  | |  | |  |
| Sarcosine | 71.62 | 100.00 | 73.22 | 7.30 |  | |  | |  |
| Serine | 83.12 | 100.00 | 36.48 | 5.99 |  | |  | |  |
| Sorbose | 117.96 | 100.00 | 77.46 | 8.10 |  | |  | |  |
| Stearic acid | 95.78 | 100.00 | 103.43 | 2.45 |  | |  | |  |
| Succinic acid | 69.73 | 100.00 | 84.14 | 9.40 |  | |  | |  |
| Succinylacetone | 76.46 | 100.00 | 94.53 | 12.10 |  | |  | |  |
| Sucrose | 79.05 | 100.00 | 58.72 | 8.88 |  | |  | |  |
| Threonic acid | 80.89 | 100.00 | 134.34 | 13.15 |  | |  | |  |
| Threonine | 131.78 | 100.00 | 93.10 | 8.70 |  | |  | |  |
| Uracil | 110.74 | 100.00 | 95.52 | 5.24 |  | |  | |  |
| Urea | 86.64 | 100.00 | 83.57 | 6.57 |  | |  | |  |
| Uridine | 113.51 | 100.00 | 80.29 | 6.11 |  | |  | |  |
| Valine | 82.02 | 100.00 | 127.95 | 6.82 |  | |  | |  |
| Liver |  |  |  |  |  | |  | |  |
| 1,5-Anhydro-glucitol | 105.82 | 100.00 | 74.44 | 5.35 |  | |  | |  |
| 2-Aminobutyric acid | 54.37 | 100.00 | 195.20 | 17.70 |  | |  | |  |
| 2-Aminoethanol | 105.64 | 100.00 | 101.16 | 3.74 |  | |  | |  |
| 2-Deoxy-glucose | 112.79 | 100.00 | 84.97 | 8.30 |  | |  | |  |
| 2-Hydroxybutyric acid | 95.78 | 100.00 | 137.29 | 7.82 |  | |  | |  |
| 2-Hydroxyglutaric acid | 114.65 | 100.00 | 68.11 | 7.38 |  | |  | |  |
| 2-Hydroxyisobutyric acid | 99.97 | 100.00 | 59.93 | 8.54 |  | |  | |  |
| 2-Ketobutyric acid | 108.64 | 100.00 | 76.68 | 6.54 |  | |  | |  |
| 2-Methyl-3-hydroxybutyric acid | 131.84 | 100.00 | 105.16 | 6.24 |  | |  | |  |
| 2-Phosphoglyceric acid | 38.35 | 100.00 | 97.97 | 10.12 |  | |  | |  |
| 3-Aminoisobutyric acid | 84.72 | 100.00 | 125.27 | 7.80 |  | |  | |  |
| 3-Aminopropanoic acid | 93.71 | 100.00 | 124.71 | 7.18 |  | |  | |  |
| 3-Hydroxybutyric acid | 86.21 | 100.00 | 104.76 | 4.72 |  | |  | |  |
| 3-Hydroxyisovaleric acid | 149.09 | 100.00 | 124.07 | 9.48 |  | |  | |  |
| 3-Methylglutaric acid | 76.59 | 100.00 | 91.80 | 17.98 |  | |  | |  |
| 3-Phosphoglyceric acid | 86.54 | 100.00 | 117.41 | 8.55 |  | |  | |  |
| 4-Aminobutyric acid | 132.17 | 100.00 | 86.16 | 8.84 |  | |  | |  |
| 4-Hydroxyphenylacetic acid | 184.69 | 100.00 | 49.60 | 18.31 |  | |  | |  |
| 4-Hydroxyproline | 86.13 | 100.00 | 132.08 | 9.59 |  | |  | |  |
| 5-Aminovaleric acid | 299.12 | 100.00 | 99.35 | 34.90 |  | |  | |  |
| 5-Oxoproline | 101.36 | 100.00 | 83.81 | 5.72 |  | |  | |  |
| Alanine | 97.87 | 100.00 | 122.21 | 5.70 |  | |  | |  |
| allo-Isoleucine | 120.16 | 100.00 | 128.63 | 8.53 |  | |  | |  |
| Arabinose | 127.04 | 100.00 | 76.95 | 6.95 |  | |  | |  |
| Arabitol | 109.99 | 100.00 | 76.27 | 7.24 |  | |  | |  |
| Ascorbic acid | 120.75 | 100.00 | 108.33 | 5.41 |  | |  | |  |
| Aspartic acid | 127.10 | 100.00 | 74.59 | 9.66 |  | |  | |  |
| Benzoic acid | 108.56 | 100.00 | 104.89 | 2.89 |  | |  | |  |
| Boric acid | 75.96 | 100.00 | 136.02 | 8.09 |  | |  | |  |
| Caproic acid | 105.71 | 100.00 | 98.30 | 1.94 |  | |  | |  |
| Citric acid | 67.31 | 100.00 | 209.59 | 28.42 |  | |  | |  |
| Cysteine | 146.09 | 100.00 | 88.30 | 20.18 |  | |  | |  |
| Decanoic acid | 88.62 | 100.00 | 88.02 | 4.58 |  | |  | |  |
| Dihydrouracil | 98.06 | 100.00 | 96.15 | 9.95 |  | |  | |  |
| Dimethylglycine | 100.74 | 100.00 | 115.92 | 9.50 |  | |  | |  |
| Ethylhydracrylic acid | 104.42 | 100.00 | 119.60 | 12.20 |  | |  | |  |
| Ethylmalonic acid | 79.72 | 100.00 | 87.17 | 11.57 |  | |  | |  |
| Fructose 1-phosphate | 338.50 | 100.00 | 61.96 | 42.76 |  | |  | |  |
| Fructose 6-phosphate | 385.61 | 100.00 | 50.07 | 52.60 |  | |  | |  |
| Fructose | 82.75 | 100.00 | 70.12 | 9.86 |  | |  | |  |
| Fumaric acid | 252.79 | 100.00 | 53.62 | 25.76 |  | |  | |  |
| Galactitol | 70.73 | 100.00 | 192.31 | 19.62 |  | |  | |  |
| Galactose | 118.91 | 100.00 | 47.08 | 11.64 |  | |  | |  |
| Gluconic acid | 145.19 | 100.00 | 83.90 | 14.54 |  | |  | |  |
| Glucono-1,4-lactone | 92.74 | 100.00 | 70.21 | 6.28 |  | |  | |  |
| Glucose | 89.53 | 100.00 | 56.88 | 16.32 |  | |  | |  |
| Glucuronic acid | 97.70 | 100.00 | 56.29 | 18.05 |  | |  | |  |
| Glutamic acid | 83.04 | 100.00 | 65.45 | 19.29 |  | |  | |  |
| Glutaric acid | 144.57 | 100.00 | 120.02 | 10.04 |  | |  | |  |
| Glyceric acid | 103.92 | 100.00 | 81.56 | 6.45 |  | |  | |  |
| Glycerol 2-phosphate | 103.82 | 100.00 | 86.00 | 4.51 |  | |  | |  |
| Glycerol 3-phosphate | 113.83 | 100.00 | 88.91 | 4.93 |  | |  | |  |
| Glycerol | 125.33 | 100.00 | 82.88 | 6.12 |  | |  | |  |
| Glycine | 111.26 | 100.00 | 92.28 | 4.31 |  | |  | |  |
| Glycolic acid | 108.61 | 100.00 | 86.58 | 5.24 |  | |  | |  |
| Glycyl-Glycine | 100.16 | 100.00 | 94.87 | 5.85 |  | |  | |  |
| Glyoxylic acid | 103.84 | 100.00 | 100.14 | 8.47 |  | |  | |  |
| Hypotaurine | 92.27 | 100.00 | 140.85 | 9.11 |  | |  | |  |
| Inositol phosphate | 94.53 | 100.00 | 111.05 | 13.32 |  | |  | |  |
| Inositol | 121.04 | 100.00 | 69.68 | 9.30 |  | |  | |  |
| Lactic acid | 100.41 | 100.00 | 96.88 | 2.36 |  | |  | |  |
| Lactose | 316.10 | 100.00 | 57.81 | 32.84 |  | |  | |  |
| Lauric acid | 106.24 | 100.00 | 91.61 | 10.03 |  | |  | |  |
| Leucine | 121.17 | 100.00 | 115.33 | 7.27 |  | |  | |  |
| Linoleic acid | 97.56 | 100.00 | 101.94 | 6.71 |  | |  | |  |
| Lyxose | 97.70 | 100.00 | 104.19 | 3.98 |  | |  | |  |
| Maleic acid | 108.06 | 100.00 | 220.66 | 37.55 |  | |  | |  |
| Malic acid | 206.83 | 100.00 | 53.44 | 21.74 |  | |  | |  |
| Mannitol | 89.64 | 100.00 | 51.22 | 11.67 |  | |  | |  |
| Mannose | 65.18 | 100.00 | 108.27 | 16.44 |  | |  | |  |
| Methylmalonic acid | 214.82 | 100.00 | 72.88 | 20.47 |  | |  | |  |
| Monostearin | 107.53 | 100.00 | 101.56 | 3.68 |  | |  | |  |
| N-Acetylglutamine | 91.15 | 100.00 | 98.02 | 8.96 |  | |  | |  |
| N-Acetylmannosamine | 72.33 | 100.00 | 91.30 | 7.21 |  | |  | |  |
| Niacinamide | 91.61 | 100.00 | 110.98 | 12.46 |  | |  | |  |
| Nonanoic acid | 99.13 | 100.00 | 104.75 | 2.95 |  | |  | |  |
| O-Phosphoethanolamine | 81.67 | 100.00 | 109.28 | 5.93 |  | |  | |  |
| Palmitic acid | 107.70 | 100.00 | 89.80 | 7.30 |  | |  | |  |
| Pantothenic acid | 110.85 | 100.00 | 89.41 | 6.31 |  | |  | |  |
| Phosphoenolpyruvic acid | 92.88 | 100.00 | 116.55 | 6.42 |  | |  | |  |
| Phosphoric acid | 108.54 | 100.00 | 93.86 | 2.81 |  | |  | |  |
| Proline | 111.18 | 100.00 | 184.12 | 14.79 |  | |  | |  |
| Putrescine | 145.43 | 100.00 | 98.73 | 15.79 |  | |  | |  |
| Pyridoxal | 104.07 | 100.00 | 95.00 | 3.67 |  | |  | |  |
| Pyruvic acid | 117.77 | 100.00 | 84.30 | 7.82 |  | |  | |  |
| Quinolinic acid | 113.61 | 100.00 | 80.63 | 7.48 |  | |  | |  |
| Rhamnose | 101.07 | 100.00 | 106.76 | 3.24 |  | |  | |  |
| Ribonic acid | 79.13 | 100.00 | 84.24 | 5.63 |  | |  | |  |
| Ribose 5-phosphate | 82.02 | 100.00 | 95.78 | 7.23 |  | |  | |  |
| Ribulose 5-phosphate | 116.56 | 100.00 | 85.74 | 9.26 |  | |  | |  |
| Ribulose | 159.51 | 100.00 | 66.50 | 12.26 |  | |  | |  |
| Serine | 141.18 | 100.00 | 69.26 | 11.41 |  | |  | |  |
| Sorbose | 185.75 | 100.00 | 69.86 | 13.56 |  | |  | |  |
| Stearic acid | 100.88 | 100.00 | 92.81 | 4.98 |  | |  | |  |
| Succinic acid | 120.11 | 100.00 | 80.40 | 7.78 |  | |  | |  |
| Sucrose | 90.07 | 100.00 | 81.87 | 5.69 |  | |  | |  |
| Threonic acid | 93.64 | 100.00 | 84.08 | 4.02 |  | |  | |  |
| Threonine | 161.01 | 100.00 | 118.63 | 9.65 |  | |  | |  |
| Uracil | 119.61 | 100.00 | 97.58 | 7.55 |  | |  | |  |
| Urea | 105.64 | 100.00 | 190.20 | 23.82 |  | |  | |  |
| Uridine | 86.69 | 100.00 | 82.77 | 6.51 |  | |  | |  |
| Valine | 124.21 | 100.00 | 116.77 | 6.20 |  | |  | |  |
|  |  |  |  |  |  | |  | |  |
| Slow-growing, intermediate-gowing , and fast-growing; growth rate duaring 1-5 days, respectively. The units are relative values. n = 6-8 in each group. | | | | | | | |  | |
|  |  |  |  |  |  |  |  |  | |
|  |  |  |  |  |  |  |  |  | |
|  |  |  |  |  |  | |  | |  |
|  |  |  |  |  |  | |  | |  |
